# Single-Cell Landscape of Mouse Islet Allograft and Syngeneic Graft

**DOI:** 10.3389/fimmu.2022.853349

**Published:** 2022-06-10

**Authors:** Pengfei Chen, Fuwen Yao, Ying Lu, Yuanzheng Peng, Shufang Zhu, Jing Deng, Zijing Wu, Jiao Chen, Kai Deng, Qi Li, Zuhui Pu, Lisha Mou

**Affiliations:** ^1^ Department of traumatic orthopedics, Shenzhen Longhua District Central Hospital, Shenzhen, China; ^2^ Department of Hepatopancreatobiliary Surgery, Shenzhen Institute of Translational Medicine, Health Science Center, The First Affiliated Hospital of Shenzhen University, Shenzhen Second People’s Hospital, Shenzhen, China; ^3^ Shenzhen Xenotransplantation Medical Engineering Research and Development Center, Shenzhen Institute of Translational Medicine, Health Science Center, The First Affiliated Hospital of Shenzhen University, Shenzhen Second People’s Hospital, Shenzhen, China; ^4^ Imaging Department, Shenzhen Institute of Translational Medicine, The First Affiliated Hospital of Shenzhen University, Shenzhen Second People’s Hospital, Shenzhen, China

**Keywords:** the immune atlas, allograft, single-cell RNA sequencing, immune heterogeneity, islet, Beta cell, islet transplantation, diabetes

## Abstract

Islet transplantation to treat the late stage of type 1 diabetic patient (T1DM) has recently made inspiring success in clinical trials. However, most patients experience a decline in islet graft function in one to three years due to immune rejection. Although the mechanisms of immune cells, including macrophages, dendritic cells (DCs), neutrophils, natural killer cells (NKs), B cells, and T cells, that mediate immune rejection have been investigated, the overall characteristics of immune infiltrates in islet allografts and syngeneic grafts remain unclear. Single-cell RNA sequencing (scRNA-seq) has provided us with new opportunities to study the complexity of the immune microenvironment in islet transplants. In the present study, we used scRNA-seq to comprehensively analyze the immune heterogeneity in the mouse model of islet transplantation. Our data revealed T lymphocytes and myeloid cells as the main immune components of grafts 7 days post-islet transplantation, especially in allografts. Moreover, our results indicated that allogeneic islet cells were transformed into antigen-presenting cell-like cells with highly expressed MHC class I molecules and genes involved in MHC class I-mediated antigen presentation. This transformation may dramatically facilitate the interaction with cytotoxic CD8^+^ T cells and promote the destruction of islet allografts. Our study provides insight into the transcriptomics and diverse microenvironment of islet grafts and their impacts on immune rejection.

## Introduction

Type 1 diabetes mellitus (T1DM) is caused by multiple factors, such as genetic and environmental factors, that lead to the autoimmune destruction of β cells (1–3). For late-stage T1DM patients, especially those with brittle diabetes, it is difficult to control various complications, such as cardiovascular disease, retinopathy, nephropathy, and life-threatening asymptomatic hypoglycemic coma, with exogenous insulin administration (4). Islet transplantation, when successful, can achieve so (5, 6). With the use of effective immunosuppressive agents, although most patients achieved insulin independence within the first year, they deteriorated as the islet graft declined rapidly afterward (7).

After islet allotransplantation, once the immune system is activated, macrophages, dendritic cells (DCs), neutrophils, natural killer cells (NKs), B cells, and T cells migrate into the graft, drive the proinflammatory cascade and destroy the graft (8, 9). As a result, the therapeutic efficacy of islet transplantation has also been largely limited by immune rejection.

Antigens of donor islet grafts, such as insulin, insulinoma-associated protein-2, glutamate decarboxylase, and zinc transporter 8, activate DCs and macrophages, which subsequently activate T cells and B cells (9). These antigens are recognized by the host immune system through the direct or indirect presentation. The direct presentation involves the immediate recognition of islet graft-derived antigen-presenting cells (APCs) and activation of host T cells. The indirect presentation involves the presentation of antigens of the graft by host APCs, thereby activating the host immune system.

After islet transplantation, CD4^+^ T cells activate the CD8^+^ T cell response, and M1 macrophages polarize and stimulate antibody production by B cells. A previous study proved that islet graft survival was prolonged when transplanted in CD4^+^ T cell knockout mice with decreased CD8^+^ T cell activity. Thus, CD8^+^ T cell-mediated immune responses play an important part in islet rejection (10–12). CD8^+^ T cells destroy islet graft cells by granule release-mediated cytolytic activities through activating Fas pathways (13, 14) and the production of IFN-γ (15). The specific attack of islet grafts by alloreactive CD8^+^ T cells also constitutes a major component of islet allograft rejection, especially by the CD103^+^ CD8^+^ T cell subpopulation (16).

The role of NKs in islet allotransplantation remains controversial. Several studies have demonstrated that liver NKs contribute to islet destruction after intraportal transplantation (17–19). In contrast, other studies proved that NKs promote islet transplantation tolerance by a perforin-dependent mechanism or by B cell-dependent tolerance (20, 21).

M1 macrophage polarization is one of the main factors contributing to the proinflammatory environment of islet grafts, which can lead to reduced graft function (22). On the other hand, M2 macrophages are anti-inflammatory (23, 24). By inhibiting the activation of M1 macrophages or promoting the activation of M2-type macrophages, the survival of islets is prolonged (22–26).

To date, the overall immune characteristics within islet grafts remain unclear. The development of single-cell RNA sequencing (scRNA-seq) has provided us with new opportunities to study the molecular characteristics of the immune microenvironment in islet transplants at the single-cell level. Moreover, scRNA-seq can identify potential cell-cell interactions by profiling receptor-ligand transcriptomics of individual cells (27, 28). To date, in the field of islet transplantation, there are no reports of immune atlases in islet grafts at single-cell resolution. Here, we used scRNA-seq to comprehensively analyze the immune heterogeneity in islet grafts and compared the transcriptome variances between syngeneic islet transplantation and allografts.

## Materials and Methods

### Animals

Wild-type 6-8 weeks old male C57BL/6 and BALB/c mice were purchased from Guangdong Medical Laboratory Animal Centre. All mice were maintained in specific pathogen-free conditions at the Central Laboratory of Shenzhen Longhua District Central Hospital. C57BL/6 mice were given a single intraperitoneal dose of 250 mg/kg body weight STZ (Sigma-Aldrich, St Louis, MO) in 0.5 M sodium citrate buffer. Two days after STZ administration, blood glucose levels were measured every day at 9:00-10:00 AM, mice with blood glucose levels steady above 16.8 mmol/L for 5 consecutive days were defined as diabetes. The animal protocols were approved by the Institutional Biomedical Research Ethics Committee of Guangdong Medical University.

### Islet Isolation and Purification

Islets were isolated as previously described (29). Briefly, we first perfused the pancreas *in situ* with 1 mg/mL Collagenase Type V (Sigma-Aldrich) *via* the common bile duct. The inflated pancreas was dissected and further digested with an additional 1 mg/mL Collagenase Type V in a 37°C water bath for 15 min. After brief vertexing, the islets were purified by discontinuous gradient centrifugation. Isolated islets were maintained in the complete CMRL-1066 medium supplemented with 10% heat-inactivated fetal bovine serum (FBS) for grafting.

### Islet Transplantation

For the syngeneic islet transplantation, 500 donor islets (C57BL/6 origin) were transplanted under the kidney capsule of one C57BL/6 recipient mice (n=5). For the allogeneic islet transplantation, 500 donor islets (BALB/c origin) were transplanted under the kidney capsule of one C57BL/6 recipient mice (n=5), as the allogeneic graft. A small incision was made at the pole of the kidney capsule. The islets were injected under the capsule through the incision using a pipette tip. The incision in the body wall was sutured.

### Graft Harvest and Dissociation of Single Cells

Seven days after transplantation, the mice were sacrificed, and the grafts adhering to the kidney capsule were harvested. The grafts were disintegrated with 0.01% (w/v) Liberase TH and 100 U/mL DNase I in RPMI 1640 for 10 min. Cells were then filtered through a 40-μm cell strainer and washed with 5 mL washing buffer (1 X PBS with 2 mM EDTA and 0.5% BSA), followed by centrifugation at 200 g for 5 min. After centrifugation, the cells were then suspended at a concentration of 1 X 10^6^ cells/mL in RPMI 1640-10% FBS and held on ice.

### Single-Cell Library Construction and Sequencing

After dissociation, the concentration of single-cell suspension was adjusted to 700–1,200 cells/μL. Cell viability was determined by trypan blue staining with a TC20 automated cell counter (Bio-rad, Hercules, CA). The ratio of viable cells was required to be more than 85%. The input cells were then loaded onto the channel of a Single Cell B Chip (v3 chemistry, PN-1000153) and loaded onto a Chromium Controller (10x Genomics, Pleasanton, CA) to generate single-cell GEMs (gel beads in the emulsion). Reverse transcription and library preparation were performed using the 1 Chromium Single Cell 3’ Reagent Kits following the 10x Genomics protocol. Libraries were sequenced, aiming at a minimum coverage of 50,000 raw reads per cell on an Illumina NovaSeq 6000 by Novogene Bioinformatics Technology Co., Ltd. (Tianjin, China).

### ScRNA-Seq Data Analysis

For syngeneic and allogeneic graft collected by our group: Sequences obtained from sequencing using the 10x Genomics single-cell RNA-sequencing platform were demultiplexed and mapped to the mm10 transcriptome using the Cell Ranger package (10x Genomics). Cells were removed if they expressed fewer than 200 unique genes, more than 4,500 unique genes, or greater than 15% mitochondrial reads. Genes not detected in at least 3 cells were removed from subsequent analysis.

To analyze the single-cell RNA-seq data, we performed Uniform Manifold Approximation and Projection for Dimension Reduction (UMAP) and t-distributed Stochastic Neighbor Embedding (t-SNE) using the Seurat R Package (version 3.1.5) with the first 75 principal components after performing the principal component analysis (PCA) on the 2000 most variable genes. Identification of significant clusters was performed using the FindClusters algorithm in the Seurat package with the resolution set as 0.6. Marker genes for each significant cluster were found using the Seurat function FindAllMarkers. Cell types were determined using a combination of marker genes identified from the literature and the CellMarker web tool (http://biocc.hrbmu.edu.cn/CellMarker/).

For public scRNA data of GSE84133-GSM2230761 and GSE84133-GSM2230762: We obtained two single-cell RNA-seq datasets of mouse pancreas (GSE84133-GSM2230761 for islets of 5 ICR mice; GSE84133-GSM2230762 for islets of 5 C57BL/6 mice) (30).

Basic filtering, classification, and visualization of the mouse pancreas dataset were performed by the Seurat R package (v3.1.5). Cells expressing fewer than 200 or more than 4,500 unique genes were filtered. The top 1000 variable genes were used for further analysis. The function FindMarkers was used based on the t-test. Ten principal components (PCs) remained for uniform Manifold Approximation and Projection for Dimension Reduction (UMAP) analysis. Cell types were identified by canonical markers.

### Cell-Cell Communication Analysis

CellPhoneDB was used to infer enriched ligand-receptor interactions among different cell types (27). Count data were used as input for the CellphoneDB algorithm (version 2.1.2) in Python 3.6. Ligand–receptor pairs are defined based on physical protein-protein interactions from the information in the CellPhoneDB website (www.CellPhoneDB.org). To identify the most relevant interactions between cell types, we looked for the cell type-specific interactions between ligands and receptors. Only interaction pairs with a p-value < 0.05 remained for the heatmap plot generated by CellPhoneDB. Chemokines, costimulatory molecules, and coinhibitory molecules were selected for visualization of interaction pairs between each two cell types. The results were visualized using the dot_plot function of CellphoneDB.

### Immunofluorescent Staining

Islet graft with the host kidney was fixed with paraformaldehyde, paraffin-embedded, and sectioned. For immunofluorescent staining, briefly, incubate the sections in two changes of xylene, dehydrate the sections with gradient ethanol, retrieve antigen in EDTA antigen retrieval buffer (pH 8.0), block endogenous peroxidase in 3% H_2_O_2_ for 25 min, block with 3% BSA at room temperature for 30 min, incubate slides with the first primary antibody overnight at 4°C in a wet box, incubate slides with the secondary antibody (respond to first primary antibody in species) at room temperature for 50 min in dark, incubate slides with TSA-FITC solution for 10 min in dark, remove the unbound primary antibodies and secondary antibodies with antigen retrieval procedure, incubate slides with the second primary antibody overnight at 4°C in a wet box, incubate slides with the CY3-labeled secondary antibody (respond to second primary antibody in species) at room temperature for 50 min in dark, quench spontaneous fluorescence for 5 min, incubate the slides with DAPI solution at room temperature for 10 min in dark, mount the slides with anti-fade mounting medium. Images were captured by Pannoramic MIDI (Hungary, 3DHISTECH).

### Statistical Analysis

For the analysis of gene expression in scRNA-seq data, all single-cell sequencing data statistical analyses were performed in the R Seurat package (3.1.5). The Wilcoxon rank-sum test was applied for comparisons in two groups. Statistical significance was accepted for *p* < 0.05.

For differentially expressed genes analyzed by the limma package in R (4.0.5), genes with a cutoff of *p*-value < 0.05 and fold change > 1.2 were determined to be differentially expressed. Heatmaps were generated by the row-scaled expression values using the pheatmap package in R (4.0.5).

## Results

### Single-Cell Analysis Reveals Dramatic Changes in Cell Heterogeneity Between Islet Allografts and Syngeneic Grafts

**Figure 1:**
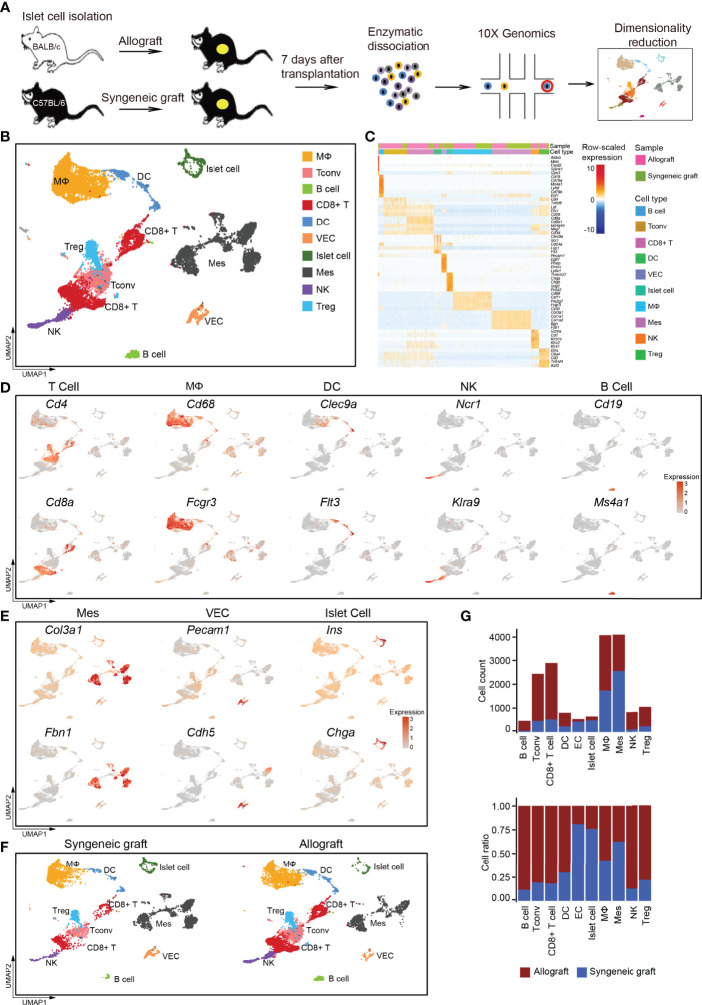
Overview of the components of cells in islet cell grafts 7 days post-transplantation by single-cell RNA-seq. **(A)** Schematic of the experimental design, single-cell sequencing, and analysis. **(B)** UMAP visualization of the total cells profiled here, with each cell colorcoded for the associated cell type. **(C)** Heatmap of row-scaled expression of marker gene expression within defined populations. Expression was measured in units of log2. **(D)** UMAP visualization shows the expression of marker genes for T cells, macrophages, dendritic cells (DCs), natural killer cells (NKs), and B cells. **(E)** UMAP visualization shows the expression of marker genes for mesenchymal cells, vascular endothelial cells, and islet cells. **(F)** UMAP visualization of the total cells from the syngeneic graft (left) and allograft (right), with each cell colorcoded for the associated cell type. **(G)** The number (upper panel) and a fraction (lower panel) of cells in the indicated cell type. Syngeneic graft n = 5, allograft n = 5. Analysis of gene expression in scRNA-seq data was performed in R using Seurat. VEC, vascular endothelial cell; Mes, mesenchymal cell; Tconv, conventional T cell.

The pretransplant blood glucose levels of transplant recipients (STZ induced diabetic mice) were higher than 20 mmol/L **(** **Supplementary Figure 1** **)**. Both syngeneic islet transplantation (C57BL/6 islets to C57BL/6 recipients) and allogeneic islet transplantation (BALB/c islets to C57BL/6 recipients) restored the blood glucose of STZ mice to normal levels from 1 to 7 days after transplantation **(** **Supplementary Figure 1A** **)**. However, immunofluorescence staining of INSULIN^+^ islet cells revealed profound destruction of islet cells in allogeneic transplantation in P14D **(** **Supplementary Figure 1B** **)**. We performed single-cell RNA sequencing (scRNA-seq) on islet allografts (BALB/c islets to C57BL/6 recipients) and syngeneic grafts (C57BL/6 islets to C57BL/6 recipients) to comprehensively identify the cell components and variations that are related to graft rejection **(** **Figure 1A** **)**. Seven days after transplantation, the grafts were harvested and subjected to 10x Genomics pipeline barcoding, library preparation, and sequencing **(** **Figure 1A** **)**. After computational quality filtering, the transcriptomes of 19,640 single cells, including 11,870 cells from allografts and 7,770 cells from syngeneic grafts, were obtained.

Using a graph-based clustering approach and the uniform manifold approximation and projection (UMAP) dimensionality reduction method in the Seurat package (31), we identified major populations of graft-infiltrated immune cells and islet cells based on their feature gene expression, including macrophages (MΦs), conventional T cells (Tconvs), B cells, CD8^+^ T cells, dendritic cells (DCs), vascular endothelial cells (VECs), islet cells, mesenchymal cells (Mes), natural killer cells (NK), and regulatory T cells (Tregs) **(** **Figures 1B, C**; **Supplementary Table 1** **)**. The major immune cell populations were composed of T cells, including CD4^+^ T cells (marker: *Cd4*), CD8^+^ T cells (marker: *Cd8a*), macrophages (MΦ, marker: *Cd68*, *Fcgr3a*), dendritic cells (DCs, markers: *Clec9a*, *Flt3*), natural killer cells (NKs, markers: *Ncr1*, *Klra9*) and a small population of B lymphocytes (markers: *Cd19*, *Ms4a1*) **(** **Figure 1D** **)**. We also captured several nonimmune cell types, including mesenchymal cells (markers: *Col3a1, Fbn1*), vascular endothelial cells (markers: *Pecam1, Cdh5*), and islet cells (markers: *Ins, Chga*) **(** **Figure 1E** **)**.

Compared with syngeneic grafts, most of the immune infiltrates in allografts were represented by T cells, macrophages, DCs, NKs, and B cells **(** **Figures 1F, G** **)**. Specifically, T cells represented the most abundant immune infiltrates in islet allografts, including CD4^+^ Tconv cells, Tregs, and CD8^+^ T cells **(** **Figure 1G** **)**. Macrophages then represented the second most abundant immune infiltrates in allografts, while NKs, DCs, and B cells were very limited in number **(** **Figures 1F, G** **)**. In contrast to immune cells that were more enriched in islet allografts, mesenchymal cells and vascular endothelial cells accumulated with the maintenance of islet cells in syngeneic grafts **(** **Figures 1F, G** **)**. To exclude the possibility that infiltrating immune cells were donor-derived tissue-resident cells, we analyzed the scRNA-seq data of healthy C57BL/6 and ICR mouse islet cells from a public database (30). These data indicated that islet-resident immune cells in baseline islet were very limited, and only a small population of macrophages was identified **(** **Supplementary Figures 2A–D** **)**. The low portion of immune cells was consistent with a previous study of human healthy pancreatic islets (32).

### T Cells Were Recruited and Activated in Islet Allograft

**Figure 2:**
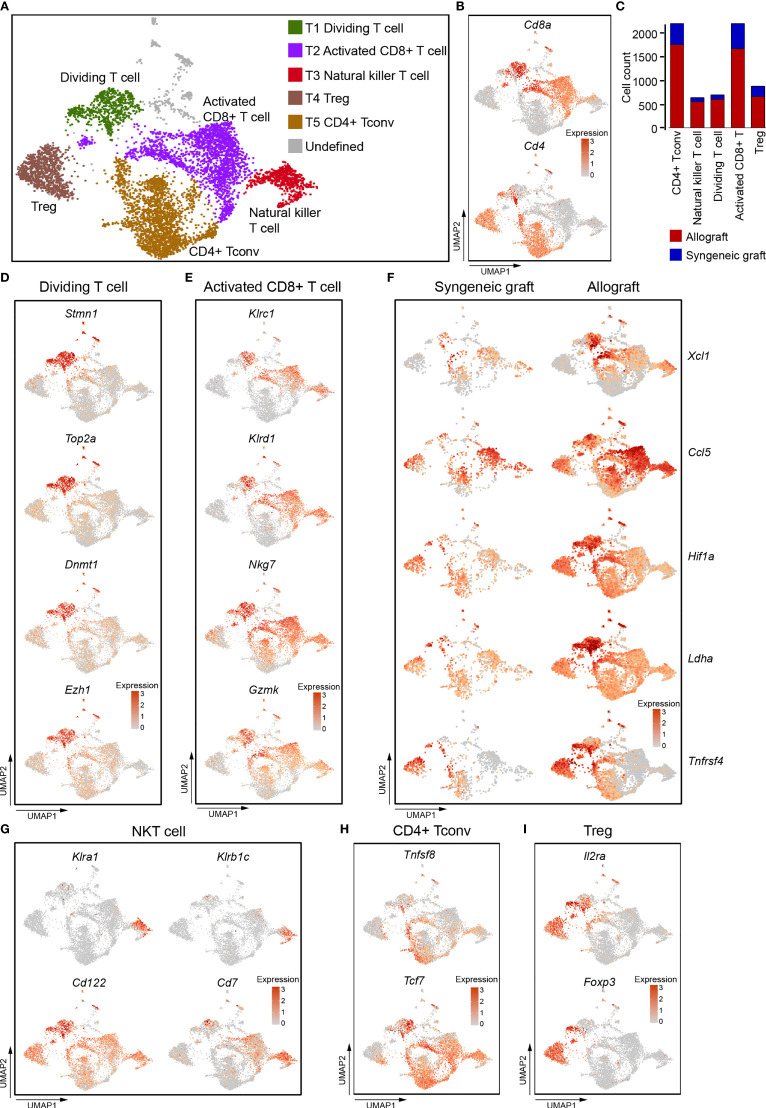
Single-cell data revealed molecular details and subclusters of T cells. **(A)** UMAP visualization of the total T cells, with each cell colorcoded for the associated cell subtype. **(B)** UMAP visualization of the distribution of CD4^+^ T cells and CD8^+^ T cells by the expression of *Cd4* and *Cd8a*. **(C)** The number of cells in the indicated cell type. **(D)** UMAP visualization shows the expression of *Stmn1, Top2A, Dnmt1, Ezh2* for dividing/proliferating T cells. **(E)** UMAP visualization shows the expression of marker genes for activated CD8^+^ T cells. **(F)** UMAP visualization shows the increased expression of *Xcl1, Ccl5, Hif1A, Ldha*, and *Tnfrsf4* in activated CD8^+^ T cells from allografts. **(G–I)** UMAP visualization of the expression of curated feature genes specific for NKT cells **(G)**, CD4 Tconv **(H)**, and Treg **(I)**. Tconv, conventional T cell; Treg, regulatory T cell.

To reveal the functional characteristics of T cells in allografts, we performed unsupervised clustering of all T cells defined in our initial analyses **(** **Figure 1B** **)**. A total of five subtypes were identified, including one cluster of cells showing high proliferating potential, one cluster of CD8^+^ T cells, one cluster of natural killer T cells, and two clusters of CD4^+^ T cells **(** **Figures 2A, B** **)**. All T cell subtypes were much more enriched in allografts than in syngeneic grafts **(** **Figure 2C** **).**

T1 cells expressed high levels of genes associated with cell division, including *Stmn1*, *Top2a*, and epigenetic regulators *Dnmt1, Ezh2* **(** **Figure 2D** **)**. These indicated that T1 cells were constantly dividing and proliferating, and that epigenetic regulation was possibly required for T cell function in acute rejection (33–35). CD8^+^ T2 cells as well as CD8^+^ T1 cells expressed natural killer cell inhibitory receptors *Klrc1, Klrd1*, and natural killer cell granule protein *Nkg7*, granular enzyme gene *Gzmk* **(** **Figure 2E** **)**, these indicated that they were activated cytotoxic cells (36, 37). It is worth noting that these cytotoxic CD8^+^ T cells, especially those from allograft, highly expressed *Xcl1 and Ccl5* **(** **Figure 2F** **)**, which are chemoattractants for blood monocytes (38, 39). CD8^+^ T1 and T2 cells from allografts also increased expression of transcription factor *Hif1a*, dehydrogenase *L*dha, and tumor necrosis factor receptor superfamily member *Tnfrsf4* **(** **Figure 2F** **)**. These genes were responsible for the proliferation and cytotoxicity of T cells (40, 41).

T3 cells did not express both *Cd4* and *Cd8* **(** **Figure 2B** **)**, but expressed *Klra1, Klrb1C, Cd122,* and *Cd7.* **(** **Figure 2G** **)**, indicating that they represented a population of CD7^+^ CD122^+^ natural killer T cells (NKT) (42, 43). They also showed an activated cytotoxic phenotype as they expressed *Klrc1,Klrd1, Nkg7,* and *Gzmk* **(** **Figure 2E** **)**. CD4^+^ T cells (T4 and T5) were composed of CD4^+^ conventional T cells (Tconv, *Tcf7*, *Tnfsf8*) **(** **Figure 2H** **)** and Treg cells (*Il2ra*, *Foxp3*) **(** **Figure 2I** **)**.

**Figure 3:**
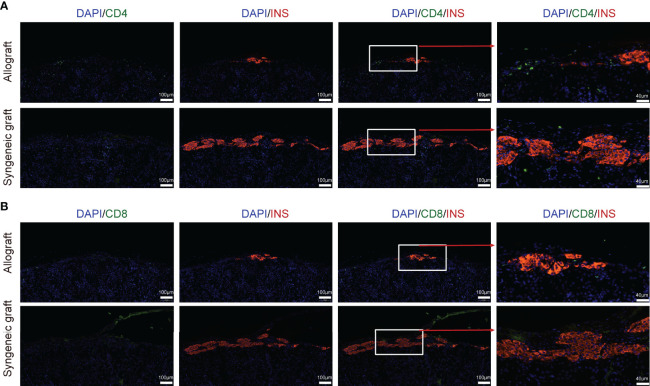
Localization of T cells in islet allograft. **(A)** Representative image of a graft section stained with antibodies specific for Insulin (red), CD4 (green), and DAPI nuclear counterstain (blue). **(B)** Representative image of a graft section stained with antibodies specific for Insulin (red), CD8 (green), and DAPI nuclear counterstain (blue). Syngeneic islets graft (C57BL/6 islets to C57BL/6 recipients) and allogeneic islets graft (BALB/c islets to C57BL/6 recipients) were harvested 7 days post-transplantation and sectioned together with mouse kidney.

To further study the molecular variations in T cells between syngeneic grafts and allografts, we analyzed their transcriptome differences. Allograft induced dramatic gene expression changes in infiltrating CD8^+^ cytotoxic T cells **(** **Figure S3A** **)**. Gene ontology (GO) pathway enrichment analysis revealed immune response and cell-cell adhesion as the top enriched signatures that differed in syngeneic grafts and allografts **(** **Figure S3B**; **Table S2** **)**. Comparison of feature gene expression between the syngeneic and allogeneic groups further identified *Ccr7*, *Cxcr3*, and *Cxcr4*, which were upregulated in allograft infiltrating CD8^+^ T cells **(** **Figure S3C** **)**. The chemokine receptor *Ccr7* is required for T cell activation in inflammation and infection (44). *Cxcr3* and *Cxcr4* are chemokine receptors that are highly expressed on effector T cells and play important roles in T cell trafficking and function (45, 46). Thus, the abundance of activated T cells in allografts indicates their function in graft rejection, and the selective inhibition of these chemokine receptors, such as *Cxcr4*, may protect the graft from immune attack (46). Consistent with the abundance of T cells from allografts in our scRNA-seq data, immunofluorescence staining of graft sections showed much more CD4^+^ T cells **(** **Figure 3A** **)** and CD8^+^ T cells **(** **Figure 3B** **)** in allograft compared with syngeneic graft.

### Macrophages Are the Main Antigen-Presenting Cells in Ectopic Islet Allograft

**Figure 4:**
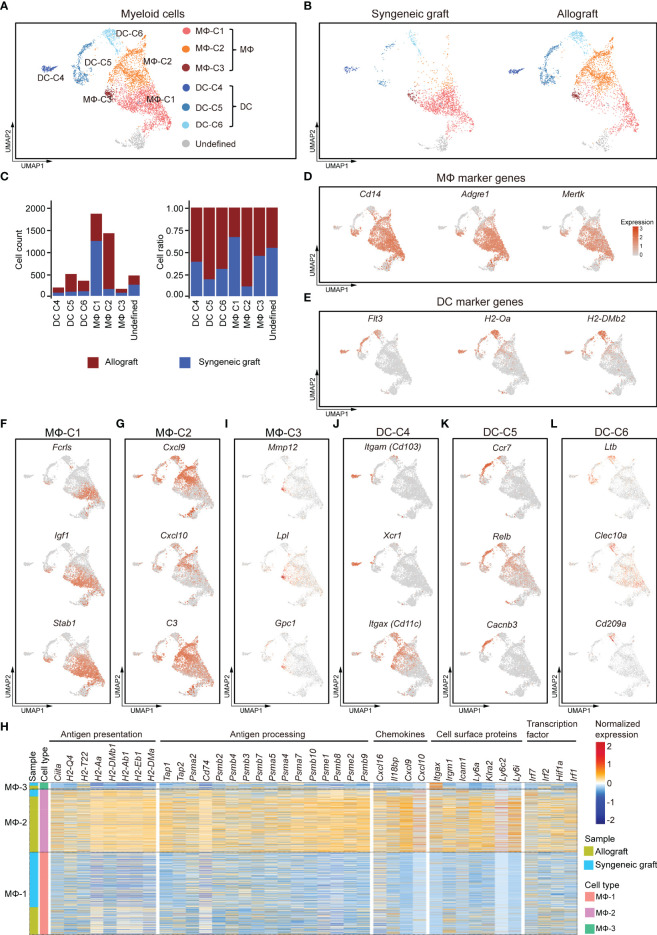
Subclusters and molecular characteristics of myeloid cells infiltrated in islet grafts. **(A)** UMAP visualization of the total myeloid cells, with each cell colorcoded for the associated cell subtype. **(B)** UMAP visualization of myeloid cells from syngeneic grafts (left) and allografts (right), with each cell colorcoded for the associated cell type. **(C)** The number (left panel) and fraction (right panel) of cells in the indicated cell type. **(D, E)** UMAP visualization shows the expression of marker genes for macrophages **(D)** and DCs **(E)**. **(F–I)** UMAP visualization of the signature genes for 3 subclusters of macrophages: MΦ-C1 **(F)**, MΦ-C2 **(G)**, and MΦ-C3 **(I)**. **(H)** Heatmap showing row-scaled expression of representative genes within 3 subclusters of macrophages. Genes were categorized by their biological functions. Expression was measured in units of log2. **(J–L)** UMAP visualization of the signature genes for 3 subclusters of DCs: DC-C4 **(J)**, DC-C5 **(K)**, DC-C6 **(L)**.

The scRNA-seq analysis of 4,286 myeloid cells that highly expressed *Cd68*, *Cd32*, and *H2-Aa* revealed six transcriptionally distinct clusters, including three clusters of macrophages (MΦ-C1, MΦ-C2, and MΦ-C3) and three clusters of DCs (DC-C4, DC-C5, and DC-C6) **(** **Figures 4A**; **Supplementary Figure 4A, B** **)**. Among these, macrophages represented the most abundant myeloid cell type. Except for cluster MΦ-C1, which contained cells mainly from syngeneic grafts, all other clusters were more frequently enriched (>60%) in allografts **(** **Figures 4B, C** **)**. Macrophages were characterized by specific expression of functional markers, including *Cd68* and *Fcgr3a* **(** **Figure 1D** **)** and *Cd14*, *Adgre1* (F4/80), and *Mertk* **(** **Figure 4D** **)**. DCs specifically expressed major histocompatibility complex class II molecules (*H2-Aa*, *H2-Dmb2*) and the cell surface tyrosine-protein kinase receptor *Flt3* **(** **Figure 4E** **)**.

MΦ-C1 was mostly enriched from syngeneic grafts **(** **Figure 4C** **)**. It was characterized by elevated expression of *Fcrls*, *Igf1*, and *Stab1* **(** **Figure 4F** **)**. *Fcrls*, which was previously found to be expressed by central nervous system-associated macrophages (47), was also highly expressed in islet graft-infiltrated MΦ subsets. M2-like macrophages secrete IGF1, which in turn regulates their activation in response to immunometabolic challenges (37). In addition, we detected higher expression of other M2-associated genes, such as *Pf4*, *Ms4a7*, *Mrc1*, *Gdf15*, *Trem2*, and *Csf1r*, in the MΦ-C1 subset **(** **Supplementary Figure 4C** **)**.

MΦ-C2 macrophages were identified as inflammatory-activated macrophages, as they highly expressed the C-X-C motif chemokines *Cxcl9*, *Cxcl10*, complement *C3*, and other M1-like macrophage marker genes **(** **Figures 4G**, **Supplementary Figure 4D**; **Supplementary Table 3** **)**. This subset mainly consisted of cells from islet allografts **(** **Figure 4C** **).** A comparison between MΦ subsets revealed an activated phenotype for the MΦ-C2 cluster with higher levels of genes involved in antigen presentation and processing, chemokine production, cell surface proteins, and transcription factors. Of note was a high expression of CIITA and MHC molecules, including class I-related *H2-Q4* and *H2-T22* and class II-related *H2-aa*, *H2-Ab1*, *H2-Eb1*, and *H2-Dma* **(** **Figure 4H** **)**. Consistently, MΦ-C2 upregulated the expression of genes related to antigen processing, including *Tap1*, *Tap2*, *Cd74*, and proteasome subunits **(** **Figure 4H** **)**. In addition, MΦ-C2 exhibited canonical inflammatory features with high expression of several chemokines (*Cxcl9*, *Cxcl10*, *Cxcl16*, and *Il18bp*), cell surface receptors (*Itgax*, *Irgm1*, *Icam1*, *Klra2*, *Ly6a*, *Ly6c2*, and *Ly6i*), and activated IFNγ signaling (*Irf1*, *Irf2*, *Irf7*, and *Hif1a*) **(** **Figure 4H** **)** (48). The MΦ-C3 population showed some common gene expression features with MΦ-C1 and MΦ-C2 and had higher expression levels of *Mmp12*, *Lpl*, and *Gpc1* **(** **Supplementary Figures 4B, I** **)**.

We identified three subpopulations of DCs (DC-C4, DC-C5, and DC-C6), although with much lower abundance than macrophages **(** **Figures 4A–C** **)**. All DC subpopulations were enriched predominantly in allografts **(** **Figure 4C** **).** DC-C4 highly expressed *Itgae* (*Cd103*), *Xcr1*, and *Itgax* (*Cd11c*), which characterized this subpopulation as CD103^+^ conventional DCs **(** **Figure 4J** **)** (49). The DC-C5 subpopulation had a CD11B^−^ CD11C^−^ phenotype **(** **Supplementary Figure 4E** **)** but highly expressed *Ccr7*, *Relb*, and *Cacnb3* **(** **Figure 4K** **),** indicating that this set represented a population of activated DCs (50, 51). The DC-C6 subpopulation was characterized by higher expression of *Ltβ*, *Clec10a*, and *Cd209a* **(** **Figure 4L** **)**. Taken together, inflammatory macrophages and DCs were enriched in islet allografts. Heterogeneity analysis revealed distinct responses of myeloid cells to xenogeneic stimuli.

**Figure 5:**
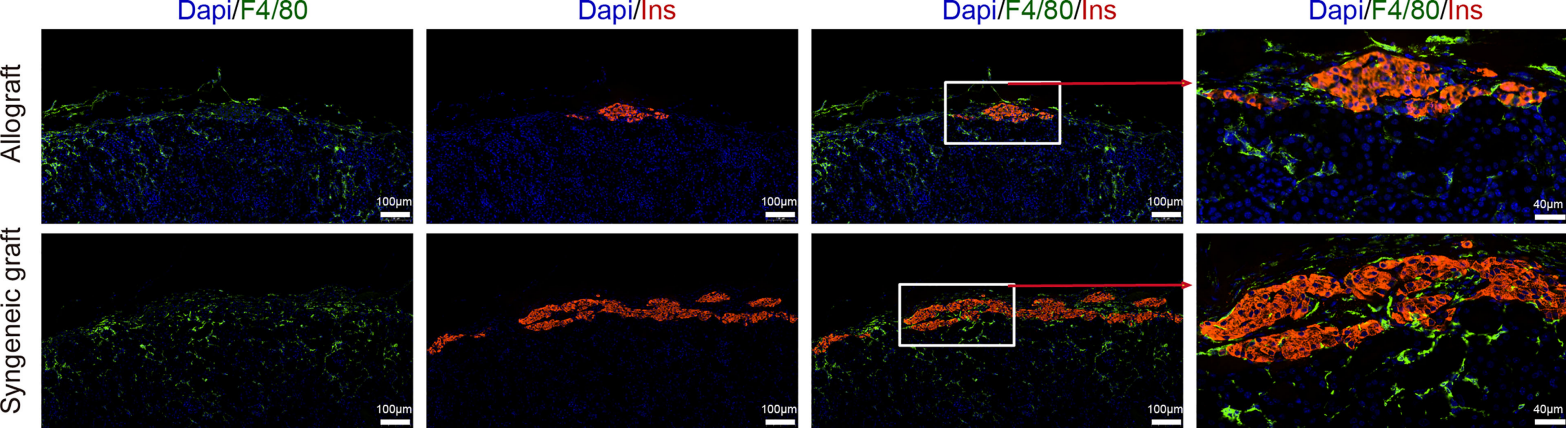
Localization of macrophages in islet grafts. Representative image of a graft section stained with antibodies specific for Insulin (red), F4/80 (green), and DAPI nuclear counterstain (blue). Syngeneic islets graft (C57BL/6 islets to C57BL/6 recipients) and allogeneic islets graft (BALB/c islets to C57BL/6 recipients) were harvested 7 days post-transplantation and sectioned together with mouse kidney.

In contrast to T cells, macrophages (including all 3 subclusters) showed similar abundance in allograft and syngeneic graft **(** **Figure 1G** **)**. Immunofluorescence staining of graft sections showed comparable macrophage infiltrates (F4/80^+^) both in allograft and syngeneic graft **(** **Figure 5** **)**.

### Islet Cells Were Activated to Facilitate CD8^+^ T Cell Interactions in Ectopic Allografts

**Figure 6:**
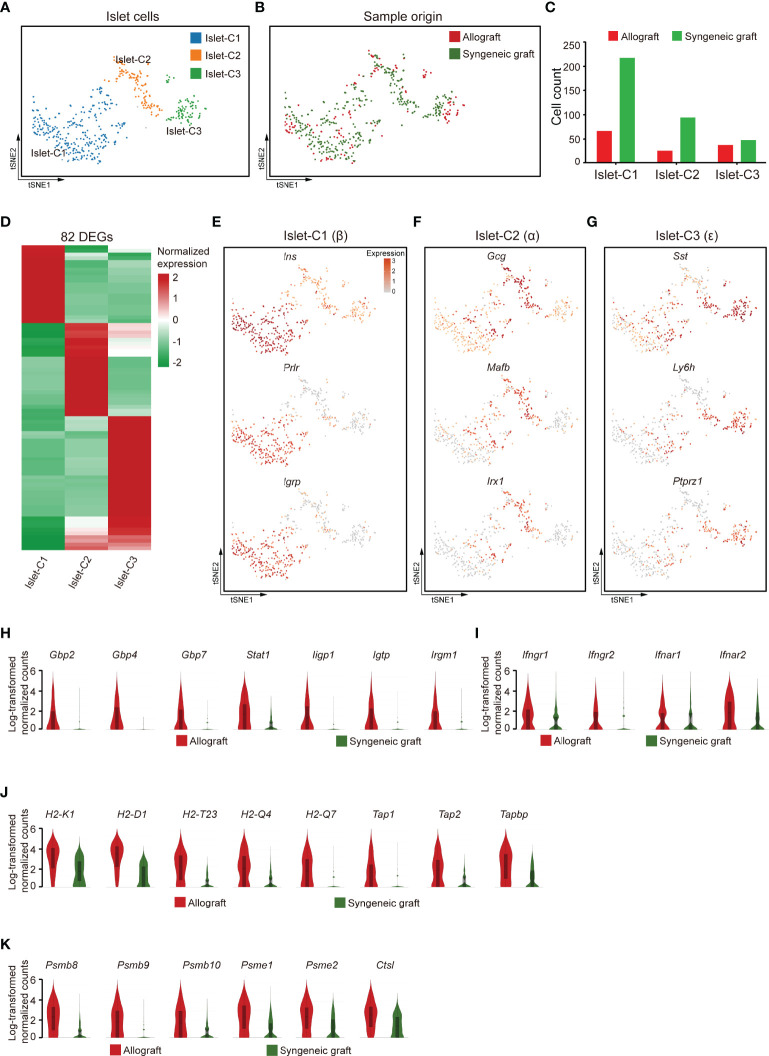
Islet cells exhibited an activated gene expression signature 7 days post-allogeneic transplantation. **(A, B)** UMAP visualization of islet cells, with each cell colorcoded for the associated cell subtype **(A)** and sample origin **(B)**. **(C)** The number of islet cells in the indicated clusters. **(D)** Heatmap showing DEG expression within 3 subclusters of islet cells. **(E–G)** t-SNE visualization of the signature genes for 3 subclusters of islet cells: islet -C1 **(E)**, islet -C2 **(F)**, islet -C3 **(G)**. **(H–K)** Violin plots showing the smoothened expression distribution of IFN-inducible genes **(H)**, receptors for interferon ligands **(I)**, MHC class I molecules **(J)**, and proteasome subunit genes **(K)** in islet cells from allografts and syngeneic grafts.

We then recovered three clusters of islet cells, most of which were obtained from syngeneic grafts, as expected because most of the islet cells in allografts were immune rejected **(** **Figures 6A–C** **)**. Based on the feature gene expression, these three clusters of islet cells were identified as β (Islet-C1), α (Islet-C2), ϵ (Islet-C3) cells **(** **Figures 6D–G** **)**. Islet-C1 was the most abundant cell subcluster and highly expressed β cell marker genes *Ins*, *Prlr*, and *Igrp* **(** **Figure 6E** **)**. Islet-C2 was a cluster of α cells that expressed marker genes *Gcg*, *Mafb*, and *Irx1* **(** **Figure 6F** **)**. Islet-C3 was identified as ϵ cells that highly expressed *Sst*, *Ly6h*, and *Ptprz1* **(** **Figure 6G** **)**.

Comparison of the gene expression features in islet cells from allografts with those from syngeneic grafts showed that IFN-inducible genes, including guanylate-binding protein (*Gbp2*, *Gbp4*, and *Gbp7*), transcription factor subunit *Stat1*, and interferon-inducible GTPase 1 (*Iigp1*, *Igtp*, and *Irgm1*), were upregulated in allograft islet cells **(** **Figure 6H** **)**. In line with the expression of IFN-inducible genes, receptors for interferon ligands, including *Ifngr1*, *Ifngr2*, *Ifnar1*, and *Ifnar2*, were also upregulated in allograft islet cells **(** **Figure 6I** **),** indicating that the islet cells in allografts were activated by interferons. Interestingly, we found that islet cells recovered from allografts highly expressed MHC class I molecules (*H2-K1*, *H2-D1*, *H2-T23*, *H2-Q4*, and *H2-Q7*) and genes involved in MHC class I-mediated antigen presentation (*Tap1*, *Tap2*, and *Tapbp*) **(** **Figure 6J** **).** In addition, islet cells from allografts also expressed a battery of proteasome subunit genes, such as *Psmb8*, *Psmb9*, *Psmb10*, *Psme1*, *Psme2*, and lysosome protease *Ctsl* **(** **Figure 6K** **)**. Together, these results indicated that islet cells in the xenogeneic microenvironment were potentially transformed into antigen-presenting cell-like cells. This transformation with high expression of MHC class I molecules specifically may facilitate the interaction of CD8^+^ T cells.

### Mesenchyme Stromal Cells Were Highly Heterogeneous in Islet Grafts

**Figure 7:**
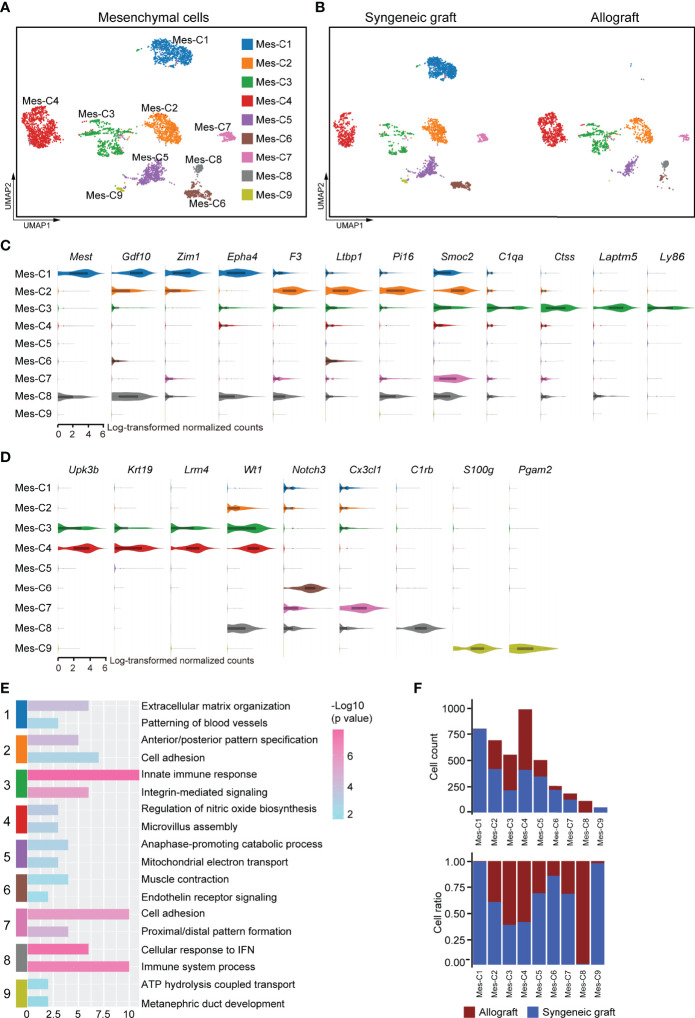
Subclusters and molecular characteristics of mesenchymal cells 7 days post-transplantation. **(A, B)** UMAP visualization of mesenchymal cells, with each cell colorcoded for the associated cell subtype **(A)** and sample origin **(B)**. **(C, D)** Violin plots showing the smoothened expression distribution of the signature genes in subclusters C1-C3 **(C)** and C4-C9 **(D)**. **(E)** The top 2 gene ontology terms were based on marker genes in mesenchymal subclusters. **(F)** The number (upper panel) and a fraction (lower panel) of cells in the indicated mesenchymal cell subclusters.

Mesenchymal cells establish a microenvironment for cell proliferation, differentiation, and immune intervention. As the transplanted islets originally contained mesenchymal cells that were recaptured in our 10x scRNA-seq dataset, we further reclustered the mesenchymal compartment. Based on the gene expression features, the mesenchymal cells were subdivided into nine transcriptionally distinct clusters **(** **Figure 7A** **)**. Among these, mesenchymal cluster 1 (Mes-C1) represented the specific enrichment of cells from syngeneic grafts **(** **Figure 7B** **)**. Mes-C1 highly expressed *Mest* **(** **Figure 7C** **)**, a negative regulator of Wnt signaling that was reported to affect neuronal differentiation (52, 53). In addition, Mes-C1 also highly expressed growth/differentiation factor 10 (*Gdf10*), transcription factor *Zim1*, and receptor tyrosine kinase *Epha4*, which may arm mesenchymal cells in an extracellular matrix organization **(** **Figure 7C** **)**. These results indicated that this subcluster of mesenchymal cells may support the reorganization of grafted islet cells. Mes-C2 was characterized by high expression of tissue factor *F3* and other extracellular signaling adaptors, including *Ltbp1*, *Smoc2*, and *Pi16*, indicating that these cells were active in cell migration and adhesion **(** **Figure 7C** **)**. Mes-C3 was more enriched in allografts and characterized by high expression of *C1qa*, *Ctss*, *Laptm5*, and *Ly86*, indicating that these cells were active in antigen processing and presentation **(** **Figure 7C** **)**. Thus, this subpopulation was possibly involved in immune responses. Mes-C4 represented another cluster that mainly originated from allografts. These cells expressed the pancreatic mesothelial cell marker genes *Upk3b*, *Krt19*, *Lrrn4*, and *Wt1* (54, 55)** (** **Figure 7D** **)**. Mes-C6 expressed *Notch3*, a receptor regulator of cell differentiation and proliferation **(** **Figure 7D** **)**. Mes-C7 cells specifically expressed the chemokine *Cx3cl1*, and Mes-C8 cells highly expressed *C1rb*, which is involved in complement activation **(** **Figure 7D** **)**. Mes-C9 highly expressed *S100g* and *Pgam2* **(** **Figure 7D** **)**. Pathway analysis identified that cell adhesion and immune responses were functionally relevant to Mes-C2, Mes-C3, Mes-C7, and Mes-C8 **(** **Figure 7E**; **Supplementary Table 4** **)**. For all mesenchymal cells, Mes-C3, Mes-C4, and Mes-C8 were much more enriched in allografts, and the remaining clusters mainly contained cells from syngeneic grafts (**Figure 7F**).

### Cell-Cell Communication in Allograft

**Figure 8:**
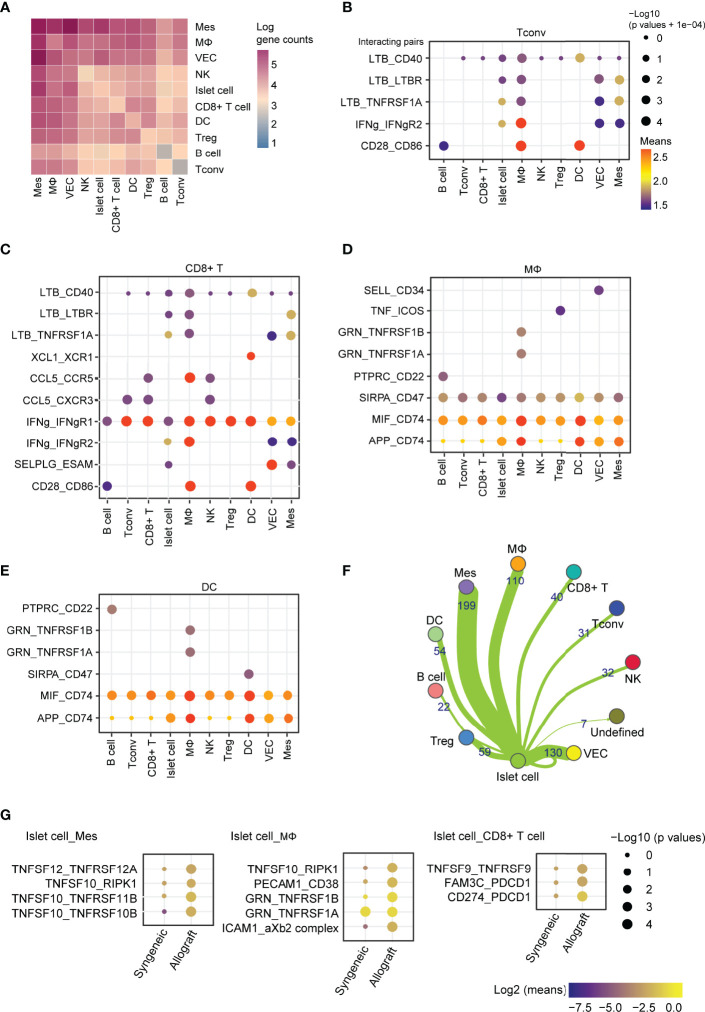
Cell-cell communication between immune cells, mesenchymal cells, and islet cells in allografts. **(A)** Heatmap showing the log of the number of interacting gene pairs involved in cell-cell communications between every two cell types. **(B–E)** Dot plot showing ligand-receptor interactions of CD4^+^ T cells **(B)**, CD8^+^ T cells **(C)**, macrophages **(D)**, and dendritic cells **(E)** with other cell types. Color represents the means of the average gene expression level of interacting molecule one in cluster one and interacting molecule two in cluster two. Cell type labels were written as follows: cell type on the panel expressing the ligand and cell type below the panel expressing the receptor. **(F)** Network map showing cell-cell communications between islet cells and other cell types based on the number of genes involved. **(G)** Dot plot showing the ligand-receptor interaction of islet cell-expressing ligands and receptors expressed by mesenchymal cells, macrophages, and CD8^+^ T cells. Color represents the maximum normalized log2 mean interaction score in each cell-cell pair, and size indicates the log of p values.

To further investigate the factors affecting the function of islet grafts, we systematically investigated ligand-receptor interactions across these cell types. Quantification of potential ligand-receptor interactions among all the cell types based on gene expression revealed strong interactions across these cell types, especially in mesenchymal cell-macrophage pairs, mesenchymal cell-islet cell pairs, Tconv-macrophage pairs, and CD8^+^ T cell-macrophage pairs **(** **Figure 8A** **)**. To further reveal the variances of ligand-receptor interactions in allografts that were possibly related to graft immune rejection, we analyzed the differentially expressed genes of selected ligand-receptor pairs in allografts. *Ltβ* and *Ifnγ* represented the most abundant ligands expressed by CD4^+^ T cells and CD8^+^ T cells for a vast range of receptors, including *Cd40*, *Ltβr*, *Tnfrsf1a*, *Ifngr1*, and *Ifngr2*, which were highly expressed by macrophages, vascular endothelial cells, islet cells, and mesenchymal cells **(** **Figures 8B, C** **)**, indicating activated T cell responses in allografts. *Ccl5* and *Xcl1* expressed by CD8^+^ T cells may also transduce signaling to macrophages and DCs through *Ccr5* and *Xcr*1, respectively **(** **Figure 8C** **)**. Macrophages and DCs showed similar cell-cell communication patterns with other cell types through GRN_TNFRSF1, MIF_CD74, and APP_CD74 **(** **Figures 8D, E** **)**, indicating an inflammatory state of these cells. Islet cells from allografts show strong interactions with mesenchymal cells and macrophages **(** **Figure 8F** **)**. Elevated expression of *Tnfsf* members *Tnfsf12*, *Tnfsf10*, and *Tnfsf9* in islet cells mediated putative ligand-receptor interactions with mesenchymal cells, macrophages, and CD8^+^ T cells **(** **Figure 8G** **)**.

## Discussion

Islet transplantation to treat the late stage of T1DM patients has recently made inspiring success in clinical trials (5, 6). However, the overall characteristics of the immune microenvironment in islet allografts remain unclear. We used scRNA-seq to comprehensively analyze the microenvironment in islet grafts using a mouse model of allografts (BALB/c islets to C57BL/6 recipients) and syngeneic grafts (C57BL/6 islets to C57BL/6 recipients). To obtain intact immune infiltrates, we abandoned any immunosuppressive agents. In mouse model of allogeneic islet transplantation, acute rejection usually occurs within two weeks posttransplant (13.8 ± 2.7 days) (56, 57). We, therefore, collected the grafts 7 days post-transplantation, because it was hard to harvest the allograft samples at later time point (14-day) (**Supplementary Figure 1B**). Our data then recovered T lymphocytes and myeloid cells as the main components of grafts 7 days post-transplantation, especially in allografts. As a matter of concern in immune cell abundance, T cells (including CD4+ and CD8+ T cells) and macrophages accounted for 58% of the total cellularity. Other immune cells, including DCs, NKs, and B cells, were detected in grafts but with low abundance.

Our results provide several novel insights into mouse islet allograft rejection. We revealed the heterogeneity of CD4^+^ T cells (Tconv and Treg) and activated cytotoxic CD8^+^ T cells, dividing T cells, and activated NKT cells. All T cell subclusters were significantly enriched in allografts compared with syngeneic grafts, consistent with previous studies showing that T lymphocytes are the main mediator of transplant rejection. Among these, CD8^+^ T cells exhibited an activated state that expressed *Ifnγ*, *Ifnβ*, and *Xcl1* and receptors *Ccr7* and *Cxcr3*. We also identified increased regulatory T cells in allografts, which may reflect feedback control of excessive immune responses in allografts. A small population of cytotoxic CD8^+^ T cells was identified in islet allografts, indicating the recruitment of host immature T cells by allogeneic islet cells. However, the function of the subpopulation needs to be further characterized.

Macrophages mediate the first phase of the immune response post-transplantation, representing the majority of cells in the transplanted organ during episodes of severe rejection (58). We detected an enrichment of M2 macrophages in syngeneic grafts with high expression of M2-associated genes such as *Igf1*, *Pf4*, *Gdf15*, *Ms4a7*, *Trem2*, *Mrc1*, and *Csf1r* in this population. These results indicated that macrophages may be required for the maintenance of exogenous islet grafts. In contrast, allograft infiltrating macrophages were inflammatory and activated, as they highly expressed the C-X-C motif chemokines *Cxcl9*, *Cxcl10*, and complement *C3*. These macrophages also expressed genes encoding molecules involved in antigen presentation and processing and chemotaxis, reflecting their roles in mediating immune responses.

As expected, we recovered rare islet cells from allografts due to immune rejection. Comparison of the gene expression features in islet cells from allografts with those from syngeneic grafts identified that the allogeneic islet cells were activated by interferons with the evidence of upregulated IFN-inducible genes and interferon ligand receptors. The high expression of *Ifnγ* by activated T cells may contribute well to the activation of allogeneic islet cells. In addition, allogeneic islet cells highly expressed MHC class I molecules and genes involved in MHC class I-mediated antigen presentation. Thus, these results indicated that allogeneic islet cells were transformed into antigen-presenting cell-like cells. This transformation may dramatically facilitate the interaction with cytotoxic CD8^+^ T cells and promote the destruction of islet allografts.

There are several limitations of this study. Firstly, we only analyzed 7 days post-transplant. Depending on the model, an additional time point later in rejection (14 or 21-day time point) would allow for analysis of memory adaptive immune responses that would better mirror those that occur in transplant rejection. Secondly, there was a lack of analysis of circulating cells to contrast with those in islets. The addition of sequencing and/or FACS on lymph nodes, spleen, and PBMCs would be important to compare with immune populations and molecules of interest to those within grafts to ensure not due to mouse strain-specific differences. Thirdly, this study only included a small number of sequenced cells and from a limited number of mice. Fourthly, only male mice were used in this study.

In summary, we revealed the microenvironment in mouse islet syngeneic grafts and allografts, including three major cell populations (T cells, macrophages, and mesenchymal cells) and five minor cell populations (DCs, NKs, B cells, VECs, and islet cells). More importantly, we identified previously unknown microenvironment variations between islet syngeneic grafts and allografts: (1) The comprehensive landscape of CD4^+^ T cells (Tconv and Treg), activated cytotoxic CD8^+^ T cells, dividing T cells and NKT cells; (2) the decreased proportion of *Fcrl* ^+^ *Igf1* ^+^ *Stabl1* ^+^ and the increased proportion of *Cxcl9* ^+^ *Cxcl10* ^+^ *C3* ^+^ macrophage subpopulation, respectively; and (3) the combined M1 and M2 gene features in macrophages of islet grafts are different from conventional M1/M2 classification. Our mouse islet microenvironment landscape provides a powerful resource for the identification of previously unknown cell subpopulations in allografts and syngeneic grafts, which suggests that these cells may contribute to the immune rejection of islet allografts.

## Data Availability Statement

The datasets presented in this study can be found in online repositories. The names of the repository/repositories and accession number(s) can be found below: Gene Expression Omnibus, accession number GSE198865.

## Ethics Statement

The animal protocols were approved by the Institutional Biomedical Research Ethics Committee of Guangdong Medical University.

## Author Contributions

PC and LM initiated the study. FY and YL performed the analysis. YP performed mouse islet transplantation and sample collection. JD prepared the figures. SZ, ZW, JC, KD, QL revised the manuscript. PC and LM wrote the manuscript. ZP, and LM designed the study and revised the manuscript. All authors contributed to the article and approved the submitted version.

## Funding

This study was supported by the Shenzhen Foundation of Science and Technology (grant numbers GJHZ20200731095207021), the National Key R&D Program of China (2017YFC1103704), and Shenzhen High-level Hospital Construction Fund (2019). This study was supported by grants from Longhua District Key Laboratory of Genomics and Precision Medicine (20170913A0410026), Longhua District Science and Technology Innovation Fund (201803, 2017006).

## Conflict of Interest

The authors declare that the research was conducted in the absence of any commercial or financial relationships that could be construed as a potential conflict of interest.

## Publisher’s Note

All claims expressed in this article are solely those of the authors and do not necessarily represent those of their affiliated organizations, or those of the publisher, the editors and the reviewers. Any product that may be evaluated in this article, or claim that may be made by its manufacturer, is not guaranteed or endorsed by the publisher.

## Supplementary Material

Supplementary Figure 1 Blood glucose levels and islets in transplanted mice. **(A)** Blood glucose levels of streptozotocin-induced C57BL/6 diabetic mice were followed for 7 days after syngeneic or allogeneic transplantation. Transplantations were performed on day 0. **(B)** Representative image of a graft section stained with antibodies specific for Insulin (red) and DAPI nuclear counterstain (blue). Syngeneic islets graft (C57BL/6 islets to C57BL/6 recipients) and allogeneic islets graft (BALB/c islets to C57BL/6 recipients) were harvested 14 days post-transplantation.

Supplementary Figure 2 Overview of the cell components in mouse islets by single-cell RNA-seq. **(A, B)** UMAP visualization of the profile of the total cell, with each cell colorcoded for the associated cell type (upper panel) and the cell count of the indicated cell type (lower panel) (**A**, GSE84133_GSM2230761, ICR islets; **B**, GSE84133_GSM2230762, C57BL/6 islets). **(C, D)** UMAP visualization shows the expression of marker genes for delta cell (*Sst*), stellate (*Rgs5*), beta-cell (*Ins*), endothelial cell (*Pecam1*), alpha cell (*Gcg*), and macrophage (*Cd68*).

Supplementary Figure 3 Gene expression signatures of T cell subclusters. **(A)** Statistical analysis of DEGs in CD8^+^ T cell, CD4^+^ Tconv, and Treg. **(B)** Gene ontology analysis of upregulated genes in each T cell subcluster. **(C)** Heatmap of row-scaled expression of marker genes for T cell activation within defined populations. Tconv, conventional T cell; Treg, regulatory T cell.

Supplementary Figure 4 Subclusters and molecular characteristics of myeloid cells infiltrated in islet grafts. **(A)** UMAP visualization shows the expression of marker genes for myeloid cells. **(B)** Heatmap showing the expression of differentially expressed genes (DEGs) within 6 subclusters of myeloid cells. **(C)** Violin plots showing the smoothened expression distribution of *Pf4*, *Ms4a7*, *Mrc1*, *Gdf15*, *Trem2*, and *Csf1r* in each macrophage subcluster. **(D)** Heatmap showing the row-scaled expression of M2 macrophage feature gene expression within 3 subclusters of macrophages. **(E)** Violin plots showing the smoothened expression distribution of *Itgae*, *Itgax*, and *Itgam* in each dendritic cell subcluster.

## Abbreviations

scRNA-seq, single-cell RNA sequencing; T1DM, type 1 diabetes mellitus; APC, antigen-presenting cells; VEC, vascular endothelial cell; Mes, mesenchymal cell; Tconv, conventional T cell; DC, dendritic cell; NK, natural killer cell; NKT, natural killer T cell; MΦ, macrophages.
