# Supplementary figures and images for: Single-Cell Landscape of Mouse Islet Allograft and Syngeneic Graft
Source: Front Immunol. 2022 Jun 10;13:853349. doi: 10.3389/fimmu.2022.853349 (PMC9226584; doi:10.3389/fimmu.2022.853349)

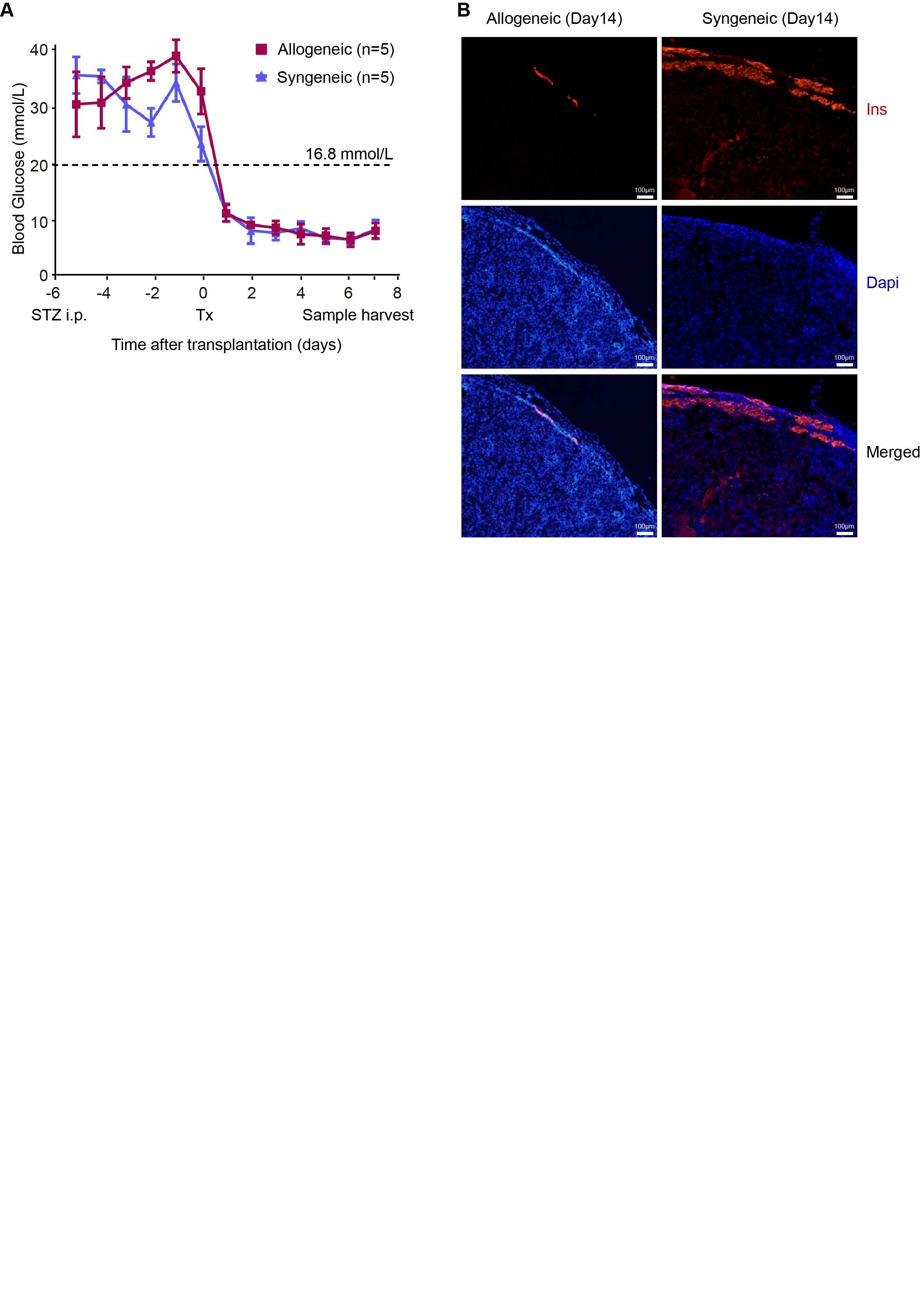

Supplement: Supplementary Figure 1 — Blood glucose levels and islets in transplanted mice. (A) Blood glucose levels of streptozotocin-induced C57BL/6 diabetic mice were followed for 7 days after syngeneic or allogeneic transplantation. Transplantations were performed on day 0. (B) Representative image of a graft section stained with antibodies specific for Insulin (red) and DAPI nuclear counterstain (blue). Syngeneic islets graft (C57BL/6 islets to C57BL/6 recipients) and allogeneic islets graft (BALB/c islets to C57BL/6 recipients) were harvested 14 days post-transplantation. [file Image_1.tif]

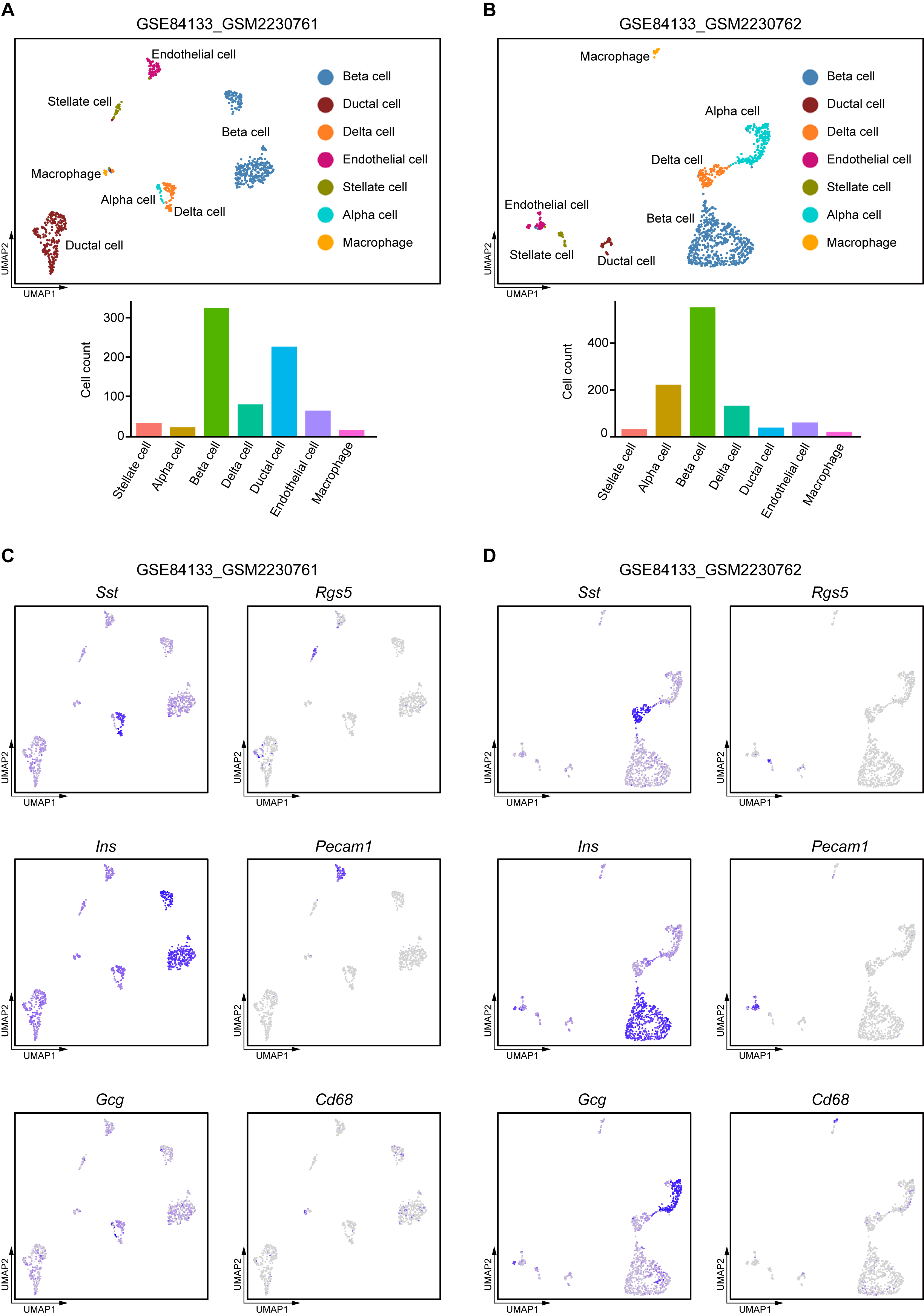

Supplement: Supplementary Figure 2 — Overview of the cell components in mouse islets by single-cell RNA-seq. (A, B) UMAP visualization of the profile of the total cell, with each cell colorcoded for the associated cell type (upper panel) and the cell count of the indicated cell type (lower panel) (A, GSE84133_GSM2230761, ICR islets; B, GSE84133_GSM2230762, C57BL/6 islets). (C, D) UMAP visualization shows the expression of marker genes for delta cell (Sst), stellate (Rgs5), beta-cell (Ins), endothelial cell (Pecam1), alpha cell (Gcg), and macrophage (Cd68). [file Image_2.tif]

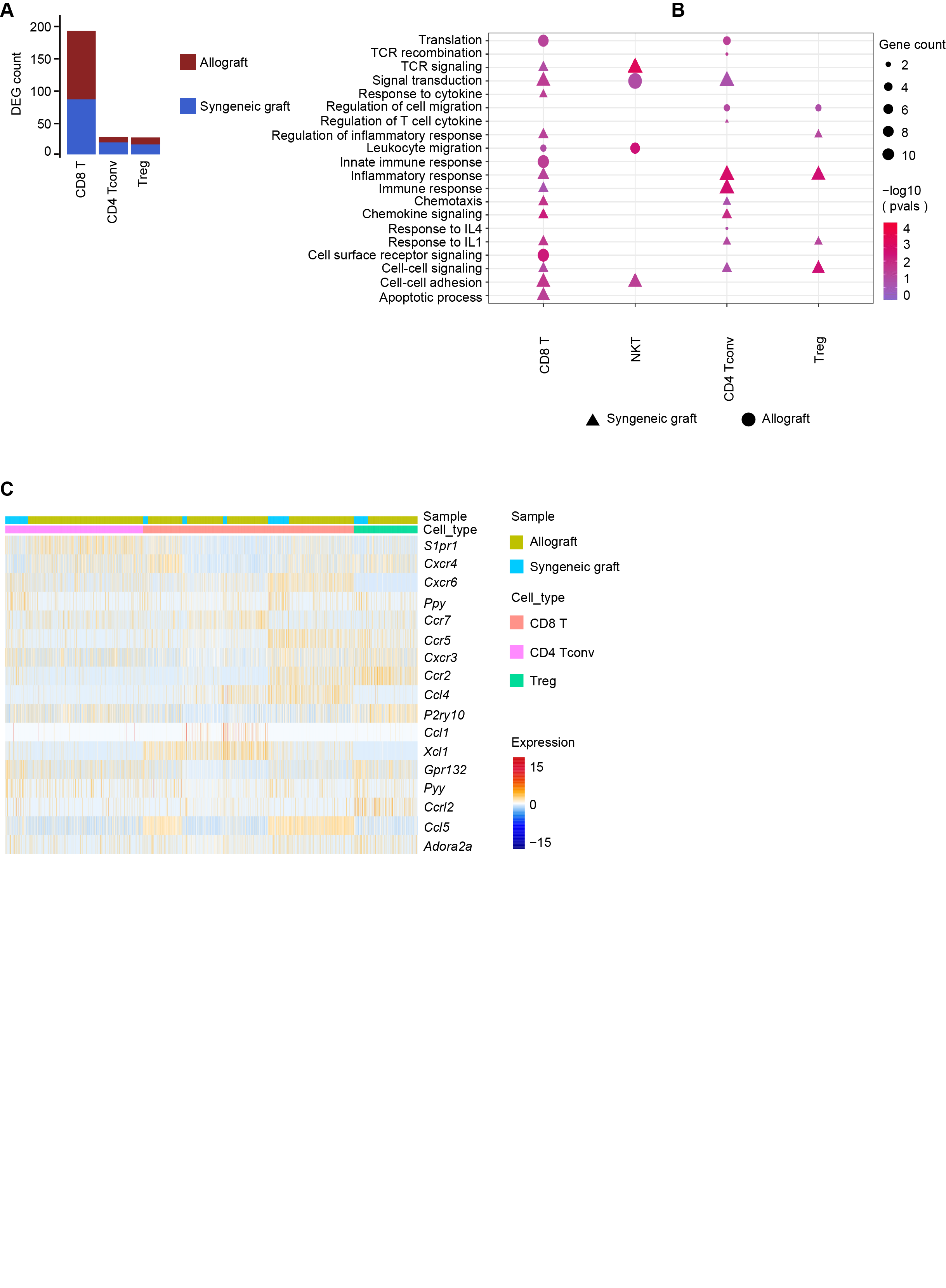

Supplement: Supplementary Figure 3 — Gene expression signatures of T cell subclusters. (A) Statistical analysis of DEGs in CD8+ T cell, CD4+ Tconv, and Treg. (B) Gene ontology analysis of upregulated genes in each T cell subcluster. (C) Heatmap of row-scaled expression of marker genes for T cell activation within defined populations. Tconv, conventional T cell; Treg, regulatory T cell. [file Image_3.tif]

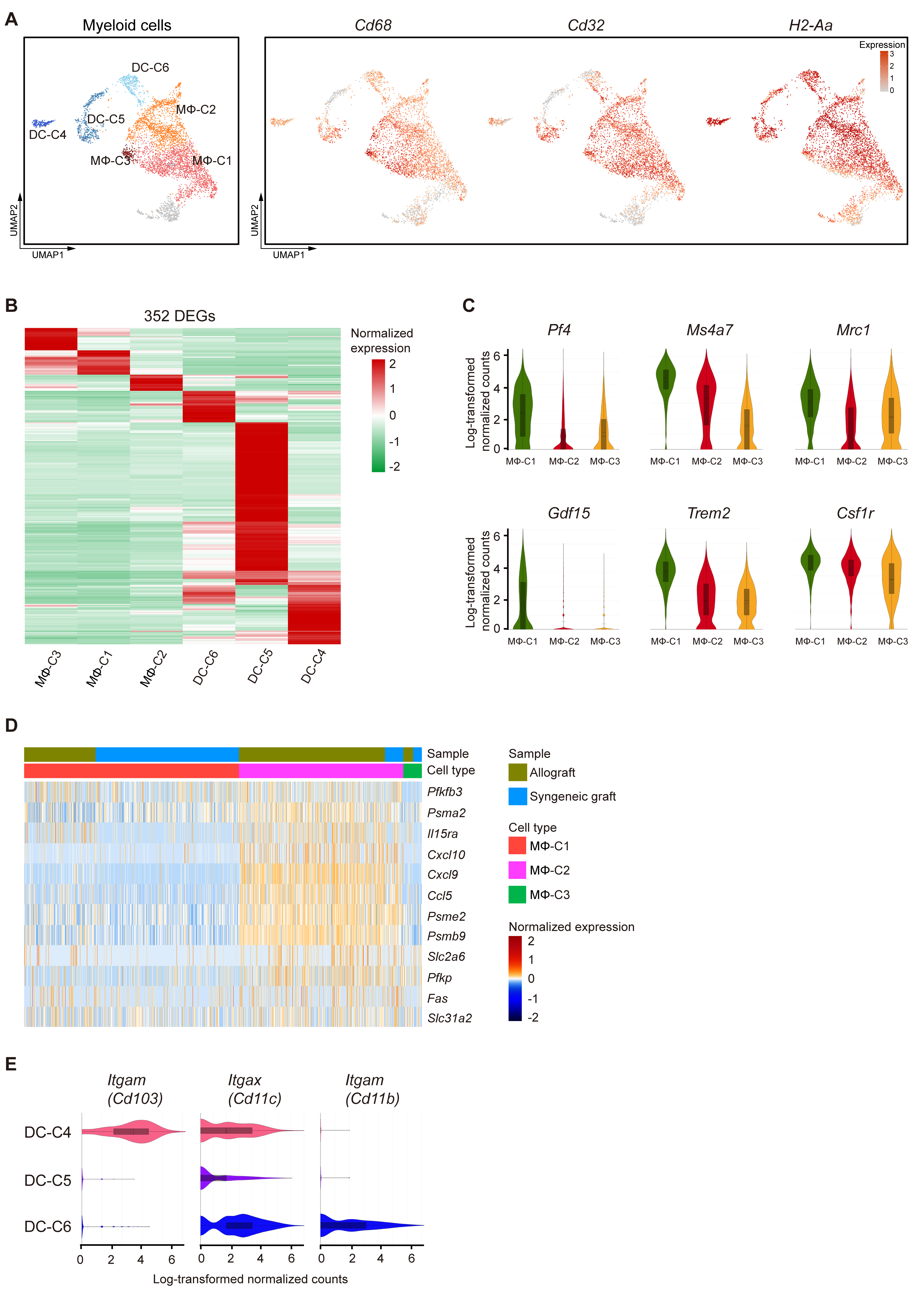

Supplement: Supplementary Figure 4 — Subclusters and molecular characteristics of myeloid cells infiltrated in islet grafts. (A) UMAP visualization shows the expression of marker genes for myeloid cells. (B) Heatmap showing the expression of differentially expressed genes (DEGs) within 6 subclusters of myeloid cells. (C) Violin plots showing the smoothened expression distribution of Pf4, Ms4a7, Mrc1, Gdf15, Trem2, and Csf1r in each macrophage subcluster. (D) Heatmap showing the row-scaled expression of M2 macrophage feature gene expression within 3 subclusters of macrophages. (E) Violin plots showing the smoothened expression distribution of Itgae, Itgax, and Itgam in each dendritic cell subcluster. [file Image_4.tif]
